# Receptor–Ligand Binding: Effect of Mechanical Factors

**DOI:** 10.3390/ijms24109062

**Published:** 2023-05-21

**Authors:** Ruotian Du, Long Li, Jing Ji, Yubo Fan

**Affiliations:** 1 Key Laboratory of Biomechanics and Mechanobiology of Ministry of Education, Beijing Advanced Innovation Center for Biomedical Engineering, School of Biological Science and Medical Engineering, Beihang University, Beijing 100191, China; 2 State Key Laboratory of Nonlinear Mechanics, Beijing Key Laboratory of Engineered Construction and Mechanobiology, Institute of Mechanics, Chinese Academy of Sciences, Beijing 100190, China

**Keywords:** receptor–ligand binding, tension, shear stress, stretch, compression, substrate stiffness, biomechanics, mechanotransduction

## Abstract

Gaining insight into the in situ receptor–ligand binding is pivotal for revealing the molecular mechanisms underlying the physiological and pathological processes and will contribute to drug discovery and biomedical application. An important issue involved is how the receptor–ligand binding responds to mechanical stimuli. This review aims to provide an overview of the current understanding of the effect of several representative mechanical factors, such as tension, shear stress, stretch, compression, and substrate stiffness on receptor–ligand binding, wherein the biomedical implications are focused. In addition, we highlight the importance of synergistic development of experimental and computational methods for fully understanding the in situ receptor–ligand binding, and further studies should focus on the coupling effects of these mechanical factors.

## 1. Introduction

The specific binding of receptor and ligand anchored on two opposing surfaces provides the molecular basis for the cell to sense, respond, and adapt to environmental cues [1], and is fundamentally important for various cellular processes such as immune response [2,3] and cancer metastasis [4,5,6]. Here, the ligand can be a small molecule or protein. Gaining insight into the two-dimensional receptor–ligand binding will contribute to uncovering numerous physiological and pathological mechanisms [7,8], as well as providing a guide for drug design [9,10,11].

In vivo, cells experience a diverse array of mechanical cues, such as tension, shear stress, stretching, compression, and substrate stiffness [12,13,14,15,16,17]. It is widely recognized that mechanical factors are essential regulators of various cellular processes, including cell adhesion, migration, growth, and differentiation [18,19,20,21,22,23], and thus are implicated in regulating relevant physiological and pathological activities [24,25,26]. The ever-developing advancement in biomechanical tools and methods further enables us to study the response of receptor–ligand binding to the mechanical stimuli at the molecular level [27,28,29,30,31,32], and new insights into the role of mechanical factors in the in situ receptor–ligand interactions are rapidly emerging [33,34]. For example, except for the ideal bonds that are insensitive to mechanical stress, two modes of mechanical regulation of binding have been proposed. Studies using the flow chamber clarified the “slip bond” [35,36], in which the lifetime of the receptor–ligand bond decreases with force [37]. Subsequently, the “catch bond” was observed with atomic force microscopy (AFM) and flow chamber experiments, showing that the bond lifetime can be increased under moderate forces in specific receptor–ligand bindings [38].

**Figure 1:**
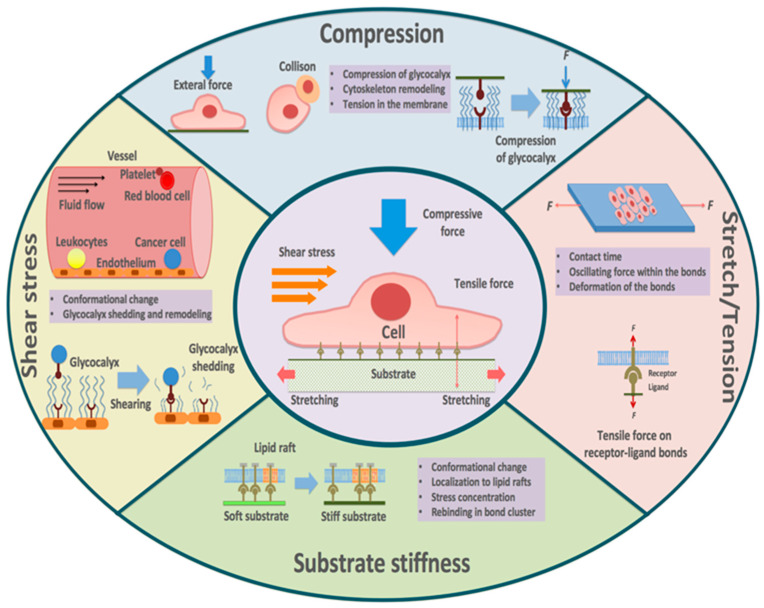
Receptor–ligand binding enduring multiple mechanical stimuli.

These results no doubt extend and deepen our understanding of receptor–ligand binding. In this review, we summarize the advances in the effects of mechanical factors on receptor–ligand binding (Figure 1), focusing on five types of mechanical stimuli: (1) tension, (2) shear stress, (3) stretch, (4) compression, and (5) substrate stiffness. Further, we highlight the biomedical implications of these mechanisms and discuss possible future research directions as well as potential new therapeutic approaches.

## 2. Characterizing and Measuring Receptor–Ligand Binding

The kinetics of receptor–ligand binding is characterized by the parameters involving kinetic rates and binding affinity. As for kinetic rates, the on-rate *k*_on_ and off-rate *k*_off_ measure the velocity of bond formation and dissociation, respectively. The binding affinity *K*_a_ = *k*_on_/*k*_off_ quantifies the binding strength of receptors and ligands [28]. In addition, the receptor–ligand bond lifetime *τ* is taken to be the inverse of the off-rate 1/*k*_off_. In contrast to the three-dimensional receptor–ligand binding in solution, the in situ binding of anchored receptor and ligand occurs in two dimensions [39,40,41], leading to the difference in the dimension of binding affinity and on-rate.

Many early experimental studies aiming at measuring the receptor–ligand binding kinetics are performed using surface plasmon resonance (SPR), which provides much enlightening information [42]. Other complementary techniques including bioluminescence resonance energy transfer (BRET) [43] and fluorescence cross-correlation spectroscopy (FCCS) [44] have also been developed to study the receptor–ligand interactions. However, these measurements cannot accurately reflect the in situ binding kinetics because of the difference in the measuring environment [45,46]. The development of experimental methods and measuring techniques has greatly advanced our understanding of in situ receptor–ligand binding [47,48]. For example, fluorescence resonance energy transfer (FRET) has been widely used as a representative fluorescence-based protocol, by which the receptor–ligand association and dissociation kinetics, as well as the binding affinity, can be directly determined by monitoring the FRET signal and fluorescent intensities [49]. Using the FRET assay, Schütz et al. found a 4–12-fold and 100-fold increase in the kinetic off-rate and affinity for the binding of T cell receptor (TCR) and peptide major histocompatibility complex (pMHC), respectively, as compared with that measured in solution using SPR [50]. Meanwhile, mechanical-based methods have also been developed to investigate the two-dimensional receptor–ligand binding [51,52,53,54,55,56]. For example, a micropipette adhesion frequency assay is utilized to measure the binding kinetic parameters by fitting the experimental data of cell–cell adhesion probability with the reaction kinetics equation, wherein the breaking event of receptor and ligand is identified by monitoring the deformation of red blood cells [57,58,59]. Another representative mechanical-based method is the flow chamber assay, which has the advantage of higher throughput and is suitable for studying the response of receptor–ligand binding kinetics to the shear stress [60,61]. Existing results indicate that the kinetic parameters measured by fluorescence-based and mechanical-based methods can differ by several orders of magnitude [62,63,64]. This intriguing unsolved issue motivates further investigations.

## 3. Tension

The receptor–ligand bonds often endure tensile forces in physiological environments. The tensile force mainly arises from the drag acting on the receptor–ligand bond due to the relative movement between the receptor and ligand molecule. This occurs in scenarios such as adjacent cells tending to be separated in response to external force [65,66,67,68]. Based on the response of receptor–ligand binding to tensile force, different types of bonds are identified as mentioned before.

**Figure 2:**
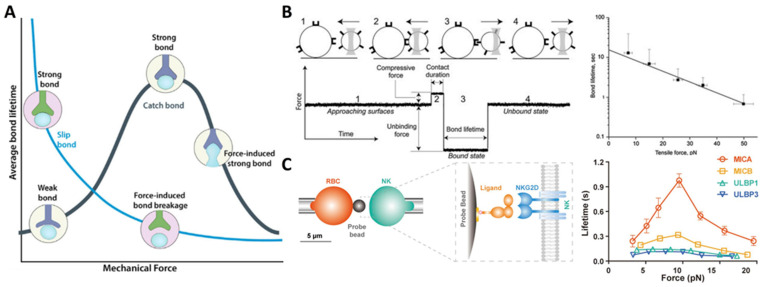
Effect of tensile force on receptor–ligand binding. (**A**) Bond lifetime decreases for slip bonds but increases for catch bonds under tension. Reprinted with permission from Changede and Sheetz [85]. (**B**) α_IIb_β_3_-fibrinogen complexes display slip-bond behavior as tensile force increases, as measured with an optical-trap-based electronic force clamp. Adapted with permission from Litvinov et al. [70]. © 2011 Biophysical Society. Published by Elsevier Inc. (**C**) Bond lifetime of NKG2D and its different ligands measured by biomembrane force probe assay. Particularly, NKG2D-MICA binding exhibits catch-slip bond behavior with increasing tensile force. Adapted with permission from Fan et al. [72]. © 2021 the authors published under the terms of the CC BY NC ND 4.0 license.

The first type is the ideal bond, which is insensitive to tensile force. Although the ideal bonds have been proposed to play a role in enabling the receptor–ligand pair to withstand tensile force, they have not yet been observed [69]. Since Bell proposed the “slip bond” in 1978, it has been widely accepted that tensile force increases the detachment rates of biological adhesive bonds [37]. For instance, by using an optical-trap-based electronic force clamp, it was found that constant tensile stress could accelerate the dissociation of integrin α_IIb_β_3_-fibrinogen [70]. Consistent with the prediction of the classical slip bond model, the average bond lifetimes exponentially decrease with increasing tensile force (Figure 2B) [70]. However, there is growing evidence that many adhesion receptors, such as selectins, counter-intuitively act in “catch bond” behavior when subjected to tensile force [38,71]. Fan et al., employing micropipette and biomembrane force probe, found that tensile force selectively prolonged the interaction lifetimes of stimulatory immunoreceptor NKG2D (natural killer group 2, member D) with certain ligands, the varying degrees of which depend on the ligand conformational changes induced by the mechanical force. More specifically, they found that tensile force induces the formation of additional hydrogen bonds at the binding interface between NKG2D and its ligand MICA (MHC class I chain-related protein A) and leads to rotational conformational changes in MICA. These findings suggest a mechano-chemical coupling mechanism that enables NKG2D to activate different immune cells in a discriminating manner for proper immune responses (Figure 2C) [72]. This catch bond behavior has also been found in the integrin–RGD (Arg-Gly-Asp) interaction. Integrin has three conformational states: bent-closed and extended-closed conformations with low affinity, and extended-open conformation with high affinity [73]. The tensile force applied to integrin suppresses its conformation fluctuations and stabilizes its active state, leading to enhanced binding affinity and prolonged bond lifetime [74,75]. In addition, Strohmeyer et al. observed a unique biphasic strengthening of binding between α_5_β_1_ integrin and fibronectin in the focal adhesion of fibroblasts in response to tensile force, where integrin-mediated cell adhesion is steeply strengthened in less than 0.5 s in the first phase, while the strengthening becomes less steep once the mechanical load exceeds a certain threshold in the second phase [76]. Two-pathway models are also proposed, wherein the receptor–ligand bond lifetime increases with tensile force as catch bond mode until a maximum value of tensile force is reached, and then the catch bond transits into classic slip bond when tensile force is further increased (that is, “catch-slip” bonds) [77,78]. For instance, Zhang et al. demonstrated a catch-slip bond transition at a force threshold in the interaction between β_3_ integrin and Kindlin2 [79]. Furthermore, a catch-slip bond transition of the interaction between leukocyte integrin macrophage-1 antigen (Mac-1) and platelet glycoprotein Ibα (GPIbα) was predicted through the dissociation probability, which provides insights into the platelet-leukocyte interactions during hemostasis and inflammatory responses under mechanical stress [80]. Interestingly, molecules of the same kind but with different conformations may respond differently to tensile stress. For instance, Rakshit et al. employed single molecule force measurements with AFM to investigate the effect of tensile force on the binding of cadherins, key molecules for maintaining tissue integrity, in two distinct conformations: X-dimer and strand-swap dimer. Results demonstrated that X-dimers formed catch-slip bonds, while strand-swap dimers formed slip bonds, which may attribute to the difference in the on-rate for the two dimers [69].

Meanwhile, researchers have described a phenomenon that the history of force application affects the strength of the receptor–ligand bond, which accumulates over repeated cycles [81]. This phenomenon is termed “cyclic mechanical reinforcement”, which occurs in bonds under the influence of cyclic tensile force. It has been demonstrated that cyclic tensile forces can induce a switch in the binding of fibronectin and integrin α_5_β_1_ from a short-lived state with a lifetime of 1 s to a long-lived state with a lifetime of 100 s. In comparison with traditional catch bonds, cycle mechanical reinforcement significantly prolongs the bond lifetime and can accumulate and persist after force removal [81]. To explain the mechanism of the switch, a three-state model has been proposed, where the receptor–ligand binding transmits among the short-lived, intermediate, and long-lived states, regulated by both loading and unloading [82]. It is noteworthy that the history of force application should also be carefully taken into account for cyclic mechanical reinforcement. Marshall et al. reported that the kinetic off-rate may rely on both the entire history of force application and the instantaneous value of force [83]. In addition, using a nanometer-scale mathematical model, Allard et al. found that the time-varying tension on the receptor–ligand bond can lead to sensitivity in bond lifetime [84].

In summary, the receptor–ligand bonds can respond differently to tensile force (Table 1), exhibiting “slip bond”, “catch bond”, or “catch-slip bond” behavior (Figure 2A), depending strongly on the type and conformation of the binding molecules. The phenomenon of cyclic mechanical reinforcement further highlights the important role of cyclic tensile force in the receptor–ligand binding.

## 4. Shear Stress

Receptor–ligand binding is involved in a variety of physiological and pathological processes (Table 2) including, for example, thrombosis, cancer metastasis, and inflammation through mediating cell adhesion, which often endures dynamic shear stress stimulation, particularly in the vasculature [86].

**Figure 3:**
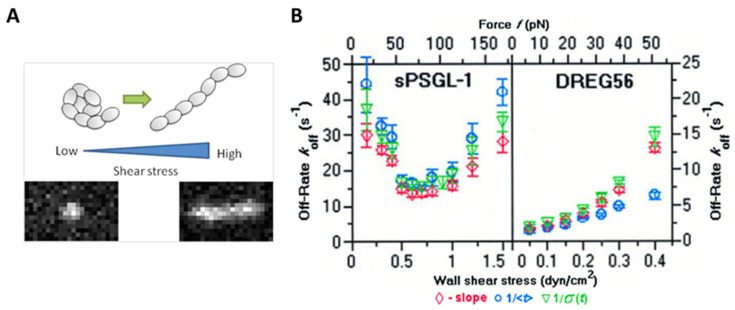
Effect of shear stress on receptor–ligand binding. (**A**) VWF undergoes a conformational transition from a compacted, globular to an extended form at high shear stress. Reprinted with permission from Vergauwe et al. [93]. Copyright © 2014 American Chemical Society. (**B**) Off-rates of L-selectin interactions with sPSGL-1 or DREG56 under shear stress. Adapted with permission from Sarangapani et al. [113].

Take for example thrombosis, which strongly depends on the erythrocyte-platelet, endothelial cell-platelet, and endothelial cell-matrix adhesion mediated via the specific receptor–ligand binding [87,88,89]. Existing results suggest that shear stress functions as a double-edged sword for blood clotting. On the one hand, the shear stress facilitates the breakage of receptor–ligand bonds. Using force spectroscopy assays, Passam et al. demonstrated that fluid shear stress enhances the fibrinogen release from integrin on the platelet surface by breaking disulfide bonds, which is detrimental to platelet adhesion and blood clotting [90]. Similarly, Wacker et al. found that fluid shear stress decreases the endothelial cell adhesion on the RGD peptides-functionalized hydrogel by regulating the integrin–RGD interaction [91]. On the other hand, shear stress can enhance the receptor–ligand binding by, for example, inducing the protein conformational change. It has been revealed that the von Willebrand factor (VWF), necessary for the platelet aggregation at the site of vascular injury, can adopt an elongated conformation at higher shear rates and expose more binding sites, which contributes to the platelet adhesion and the platelet plug formation (Figure 3A) [92,93,94]. In addition, the formation of disulfide bonds on VWF is shown to be promoted in response to shear stress, thereby further enhancing the binding of VWF to platelets [95].

In the process of hematogenous or lymphatic metastasis, the shear stress generated by the bloodstream or lymph flow has also been proven to play an important role in affecting the binding of receptors on tumor cell membranes with their ligands, which mediates the adhesion of tumor cells to tissues such as blood vessels and lymph nodes [96,97,98,99]. It has been shown that a certain range of shear stress is required for the adhesion of cancer cells during metastasis. For example, Gomes et al. found that breast cancer cells adhere most to vein endothelial cells under low shear stress compared with static conditions [100]. Spencer et al. observed an increase in the adhesion of breast cancer cells to collagens and fibronectin at moderate shear stress levels compared with static conditions or other shear levels [101]. Similar results are obtained for the β_1_ integrin-mediated binding of cancer cells to laminin, an extracellular matrix (ECM) component within the lymph node parenchyma, in response to shear stress induced by lymphodynamic flow [102]. Additionally, hemodynamic shear stress can also regulate receptor–ligand binding and cancer cell adhesion by affecting glycocalyx shedding and remodeling. As an exterior cell surface layer, the glycocalyx is thicker than most adhesion receptors and thus prevents the specific binding of receptors and ligands. Therefore, the glycocalyx is often considered a barrier to cancer cell adhesion [103,104]. Experimental results suggest that shear stress stimulus can alter the molecular composition and thickness of the glycocalyx, allowing more available receptors to bind with adhesion ligands on cancer cells [99,105,106,107,108]. Moreover, the receptor–ligand binding shows a shearing direction-dependent manner, because the shear stress-induced force acting on the receptor–ligand bond can regulate the, for example, protein conformational change, depending on the applied force value and direction [101].

In the context of the inflammatory response, the tethering and rolling of leukocytes on vascular surfaces are highly regulated by shear stress through the interactions of adhesion proteins such as selectins with their ligands [109,110]. Selectin–ligand bonds have high binding strength, which contributes to the initial tethering to the vessel wall. Meanwhile, the fast on- and off-rates of the selectin–ligand bonds facilitate rolling when responding to hydrodynamic drag. Early flow chamber experiments demonstrated that the off-rates of L–selectin interactions with ligands such as P-selectin glycoprotein ligand-1 (PSGL-1) increased with wall shear stress [35,111,112]. However, subsequent evidence suggests that the off-rate of L-selectin ligand bindings decreases with increasing the applied force acting on the receptor–ligand bond at low shear stress (that is, catch-bond behavior), but increases with increasing applied force at high shear stress (that is, slip bond behavior) (Figure 3B) [113]. Correspondingly, using techniques such as AFM, researchers have observed a shear threshold effect, which indicates that cell rolling requires a certain level of shearing [113,114,115,116]. The responses of on-rate and off-rate to the dynamic shearing are thought to be responsible for the shear threshold phenomenon. A minimum shear is required to support rolling and to enhance the overall on-rate. When the shear rate reaches the minimum threshold level, selectin receptor–ligand binding exhibits “catch bond” behavior, and the binding is continually strengthened as the applied force increases. As the applied force increases further, however, receptor–ligand binding is converted to “slip bond” behavior, which means that higher shear stress accelerates bond dissociation [113,117,118]. The shear threshold effect is believed to arise from a delicate balance between the adhesive force of receptor–ligand binding and the dispersive hydrodynamic force. To further interpret the phenomenon, researchers have analyzed the structure of the interacting molecules. Their results showed that L-selectin can present an extended conformation with high affinity in the presence of applied force, in comparison with a bent conformation with low affinity in the absence of applied force, due to its flexible hinge region [119].

Overall, shear stress indeed plays a critical role in regulating receptor–ligand binding and cell adhesion-related physiological and pathological functions. Whether the effect of shear stress is positive or negative depends on several factors, such as the direction and intensity of the shear stress, the type of proteins involved, and the specific cell types affected. In addition, the shear stress should be controlled within a reasonable range because high shear stress can reduce cell viability [120,121].

**Table 2:** Relevant studies regarding the effect of shear stress on receptor–ligand binding.

Biomedical Implications	Molecules	Shear Stress	Author [Reference]
Thrombosis	Integrin α_IIb_β_3_ and intercellular adhesion molecule-4 (ICAM-4)	Mainly occurs at shear rate below 300 s^−1^	Du et al. [87]
	Integrin α_IIb_β_3_ and fibrinogen	1000 s^−1^ and 3000 s^−1^	Passam et al. [90]
	Integrin and RGD	20 dyn/cm^2^	Wacker et al. [91]
	VWF and collagen	10^0^–10^5^ s^−1^	Schneider et al. [92]
	VWF and collagen	—	Wei et al. [94]
	VWF and GPIbα	50 and 100 dyn/cm^2^	Choi et al. [95]
Cancer metastasis	Collagens, vitronectin, and fibronectin	0.5, 1, 2, and 3 dyn/cm^2^	Spencer and Baker [101]
	β_1_ integrins and laminin	0.07 dyn/cm^2^	Fennewald et al. [102]
	L-selectin and nucleolin	0.07 dyn/cm^2^ (~8 s^−1^)	Goldson et al. [122]
Inflammatory response	L-selectin and carbohydrate ligand	0.3–2 dyn/cm^2^	Alon et al. [35]
	L-selectin and PSGL-1	0.5–2 dyn/cm^2^	Ramachandran et al. [111]
	L-selectin and peripheral node addressin (PNAd)	0.5–2 dyn/cm^2^	Smith et al. [112]
	L-selectin and PSGL-1	0.15–1.5 dyn/cm^2^	Sarangapani et al. [113]
	L-selectin and PSGL-1	0–300 s^−1^	Caputo et al. [114]
	L-selectin and PNAd	0–10 dyn/cm^2^	Finger et al. [115]
	L-selectin and PSGL-1	10^1^–10^4^ s^−1^	Yago et al. [116]
	L-selectin and PNAd	0.4–4.0 dyn/cm^2^	Lawrence et al. [118]

## 5. Stretch

**Figure 4:**
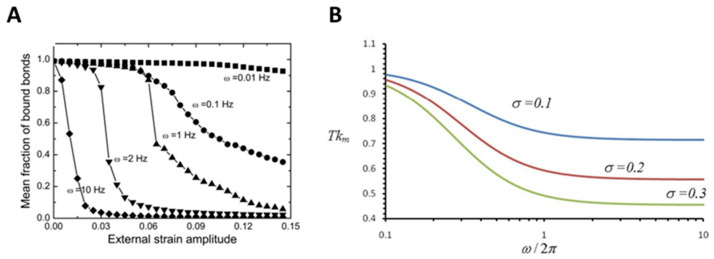
Effect of stretch on receptor–ligand binding. (**A**) Mean fraction of receptor–ligand bond as a function of the external strain at different frequencies. Adapted with permission from Kong et al. [133]. © 2008 The Biophysical Society. Published by Elsevier Inc. (**B**) Average lifetime of catch bond as a function of cyclic frequency. Adapted with permission from Chen et al. [135].

Mechanical stretch, resulting from, for example, the pulsatile nature of blood flow and ECM perturbations, has long been recognized as a fundamental force stimulus affecting cellular functions [123,124,125]. As a typical example, the mechanical stretch has been proven to play a critical role in modulating cell reorientation, wherein cell bodies realign nearly perpendicular to the stretching direction [126,127,128,129,130,131]. The development of methods and technologies enables researchers to further study the molecular mechanism underlying the effect of the mechanical stretch on cellular functions (Table 3). For example, to further understand the stretch-regulated cell reorientation, many studies have focused on the receptor–ligand binding for cells cultured on, for example, a cyclically stretched substrate, mimicking the mechanical stretch. Qian et al. developed a mechanochemical modeling framework to investigate the reorientation of spindle-shaped cells under cyclic stretch by considering the dynamic evolutions of adhesive receptor–ligand bond clusters. They demonstrated that the final alignment of cells under stretching is affected by the balance between the growth and disruption of cell-substrate adhesion regulated by receptor–ligand binding in a stretching frequency and amplitude-dependent manner [132]. Kong et al. developed a focal adhesion model at the molecular level, which takes into account the contribution of receptor–ligand binding. Their results indicated that mechanical stretch at a frequency beyond a threshold value would cause the disruption of the receptor–ligand bond cluster due to the short contact time between receptors and ligands, or the deformation of the receptor–ligand bonds in the adhesion cluster induced by the stress fiber stiffening (Figure 4A) [133]. Further, in view of the important role of the catch bond (for example, integrin–ligand bond) in determining the strength of focal adhesion connecting the cell and the substrate [134], Chen et al. have shown that the force within the catch bond undergoes periodic oscillations during the cyclic stretch, and the amplitude of this force oscillation increases with the stretching amplitude and frequency. According to their analysis, a larger amplitude of force variation within the catch bonds reduces the bond lifetime, which in turn destabilizes the focal adhesions. This would lead to the slide or relocation of focal adhesions and then cause the associated stress fibers to contract and rotate to the most stable configurations (Figure 4B) [135]. It is hypothesized that cells tend to orient themselves in the direction where the maximum bond densities are achieved to realize the strongest cell-substrate attachment [132].

These results highlight the important role of mechanical stretch in receptor–ligand binding and focal adhesion, which are shown to be responsible for stretch-regulated cellular functions. Meanwhile, these findings should also be meaningful for improving our knowledge of angiogenesis and other diseases associated with blood vessels and the heart, because cyclic deformation is a common physiological condition in these systems [136].

## 6. Compression

**Figure 5:**
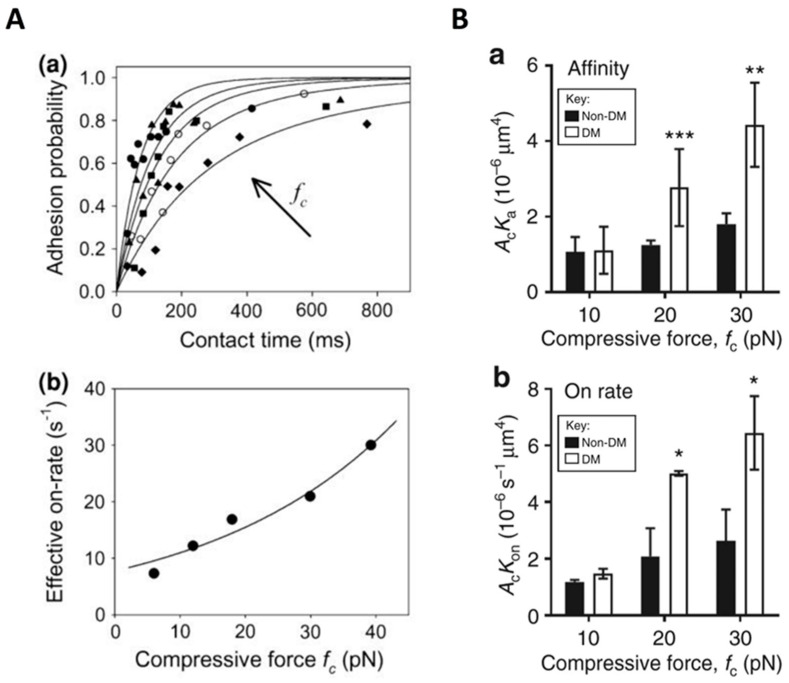
Effect of compressive force on receptor–ligand binding. (**A**) Adhesion probability and effective on-rate of the interaction between E-selectin and sialyl Lewis^a^ as a function of compressive force. Reprinted with permission from Snook and Guilford [144]. Copyright © 2012, Biomedical Engineering Society (**B**) Effective binding affinity of integrin α_IIb_β_3_ and fibrinogen in diabetic platelets from non-diabetic (non-DM) and diabetic (DM) mice in response to compressive forces. * *p* < 0.5; ** *p* < 0.01; *** *p* < 0.001. Adapted with permission from Ju et al. [145].

Compression is an essential factor in the mechanical microenvironment of cells and can be generated by cell–cell collision or external force (for example, applied pressure on the skin) [137,138,139]. Intuitively, compression tends to decrease inter-membrane separation. For a simplified adhesion system with only membrane-anchored receptors and ligands, the receptor–ligand binding affinity is found to be significantly reduced in the presence of compression [140], partially due to the changed separation of receptor–ligand binding sites. In addition to the specific binders, the cells are also covered with the glycocalyx layer. The thickness of the glycocalyx layer ranges from tens to hundreds of nanometers and is generally larger than the length of the receptor–ligand bond, thus imposing a detrimental effect on the specific binding of receptors and ligands and leading to decreased binding affinity [62]. Introducing compression will compress the glycocalyx and contribute to the exposure of the binding site of receptor and ligand, thereby facilitating their binding [141,142]. Using the thermal fluctuation assay, Snook and Guilford observed an increased on-rate of the binding of E-selectin with sugar on PSGL-1 called sialyl Lewis^a^ under compressive forces and provided single molecular evidence that compressive load affects not only the off-rate but also the on-rate of the receptor–ligand binding [143]. Subsequently, they utilized a magnetic bond puller to demonstrate the compressive load-dependent rate of bond formation between E-selectin and the sugar on PSGL-1. They also found that these two molecules could form a catch-slip bond. Similar to their previous study, the on-rates increased with increasing compressive force. Although the average magnitudes of the on-rates were approximately 2-fold lower than those determined with the thermal fluctuation assay, their dependence on the compressive force is comparable (Figure 5A) [144]. In addition, Ju et al. conducted a study utilizing a biomembrane force probe and found that compressive force promotes the affinity maturation of integrin α_IIb_β_3_ on discoid diabetic platelets and increases integrin–fibrinogen association rate (Figure 5B) [145]. To explain this effect of compressive force on the integrin–fibrinogen binding, they proposed that, on the one hand, the induced tension in the membrane due to compressive force may trigger the opening of Ca^2+^ channels and lead to integrin activation; on the other hand, the external compressive force may cause the remodeling of the platelet cytoskeleton, leading to integrin activation [145]. The findings mentioned above provide insight into the role of compressive force in receptor–ligand binding (Table 4), and further studies on how the compressive force affects receptor–ligand binding are needed.

## 7. Substrate Stiffness

In addition to the aforementioned types of forces, the mechanical properties of the substrate are also important mechanical factors that affect receptor–ligand binding (Table 5). It has been confirmed that tissue stiffness can change with aging [146] or pathological conditions [147,148], which in turn leads to cellular response. Typically, cells establish more stable adhesion on stiffer substrates [149,150,151,152,153,154,155] and can exhibit positive or negative durotaxis behavior [156,157]. As the molecular basis of cell adhesion and migration, the two-dimensional receptor–ligand binding has also been proven to be regulated by the substrate stiffness in both physiological and pathological processes, such as inflammatory and immune response, stem cell differentiation, and cancer.

In recent years, there has been growing evidence that substrate stiffness affects receptor–ligand bindings during inflammatory processes. As mentioned above, leukocyte rolling along the endothelium is primarily mediated by P-, E-, and L-selectins and their complementary ligands [158]. MacKay and Hammer measured the rolling velocity and capturing efficiency of monocytic cells perfused over E-selectin-functionalized or P-selectin-functionalized hydrogels with different stiffness. Their results showed that the attachment through E-selectin was enhanced on stiffer gels, while cell attachment to P-selectin-coated gels was independent of substrate stiffness [159]. Consistent with this experimental observation, Moshaei et al. examined how substrate stiffness modulates cell adhesion and kinetics and discovered that the trajectory of rolling cells on E-selectin-coated substrates was sensitive to the substrate stiffness while that on P-selectin-coated substrates was insensitive [160]. This difference may be attributed to the higher energetic affinity of P-selectin to the leukocyte ligands [159,160,161]. Further, Wu et al. carried out a micropipette adhesion frequency assay and found that stiffening the carrier lowered the binding affinity of P-selectin and PSGL-1 by reducing the forward rate, while the opposite is true for softening the carrier [162]. These findings are important for understanding the mechanisms of leukocytes rolling on and tethering to endothelial cells in physiological and pathological processes. In addition, modeling results indicate that the cell migration velocity differs for multiple types of integrins with different binding kinetics in response to the substrate stiffness, suggesting that the existence of different integrins with varied binding kinetics functions as an adaptation mechanism for substrate stiffness [163].

**Figure 6:**
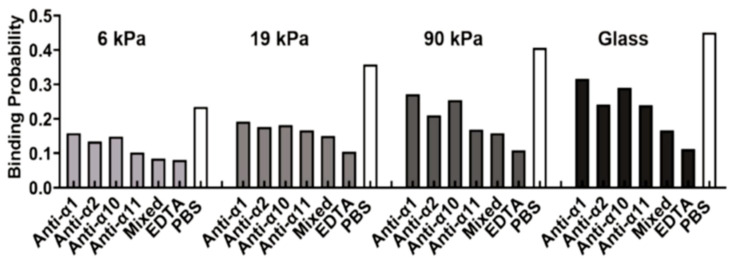
Effect of substrate stiffness on receptor–ligand binding, showing the binding probability between collagen and integrins for SiHa cells grown on a substrate with different stiffness. Adapted with permission from Zhuang et al. [166]. Copyright © 2022 Elsevier Inc.

The role of substrate stiffness in receptor–ligand bindings during stem cell differentiation has also been revealed. Experimental results showed that the binding of integrins to their ligands (for example, peptide, collagen, fibronectin) is enhanced and mesenchymal stem cells have higher cell attachment on the relatively stiffer substrates potentially due to the induced change in adhesion bonds state (tensioned or relaxed) and integrin conformational stability, contributing to a better understanding of their differentiation in a substrate-dependent manner [164,165]. A similar stiffness response has also been observed for the integrin-regulated adhesion of cervical cancer cells on the substrate (Figure 6) [166]. In addition to integrin bonds, stiffness-dependent behavior is also observed for the interaction of vinculin and its ligands. Nagasato et al. found that rigid substrates promoted vinculin binding to vinexin α, leading to a vinculin conformational change to its activated form with reduced head-to-tail association, and redistribution to lipid rafts, as well as the stable localization of vinculin at focal adhesions [167]. These findings provide insights into the regulation of stem cell differentiation by substrate stiffness [167].

There are many other studies performed to investigate the effect of substrate stiffness on receptor–ligand binding [168,169]. Their results further provide insight into the underlying mechanism involving, for example, cytoskeleton and binding cooperativity [168,169]. In practice, the substrate stiffness may serve as a potential regulatory target for regulating receptor–ligand binding and cellular functions. Therefore, further in-depth investigations are needed to offer a basis and reference for the application.

**Table 5:** Relevant studies regarding the effect of substrate stiffness on receptor–ligand binding.

Biomedical Implications	Molecules	Substrate Stiffness	Author [Reference]
Inflammatory response	E-selectin, P-selectin	1, 5, 10, 24, and 84 kPa	MacKay and Hammer [159]
	E-selectin, P-selectin	1, 10, and 100 kPa	Moshaei et al. [160]
	P-selectin and PSGL-1	Stiffness and microtopology of three carriers	Wu et al. [162]
	Integrin	1–20 kPa	Feng et al. [163]
Stem cell differentiation	Integrin α_5_β_1_ and peptide ligand	~2 and ~25 kPa	Gandavarapu et al. [164]
	β_1_ integrin	9, 25, and 48 kPa	Gershlak and Black [165]
	Vinculin and vinexin α	2.6 and 34 kPa	Nagasato et al. [167]
Cancer	Integrin and collagen	6, 19, 90 kPa, and glass	Zhuang et al. [166]

## 8. Conclusions and Future Prospectives

Understanding the mechanosensing and mechanotransduction processes implicated in various physiological and pathological processes and how they affect cell viability, protein expression, and function are of paramount relevance. Elucidating the responses of receptor–ligand bindings to the mechanical microenvironments will contribute to the pharmaceutical and biomedical fields. It has been revealed that various mechanical factors, such as tension, shear stress, stretch, compression, and substrate stiffness play a crucial role in mediating receptor–ligand binding. Here, we review the contribution of these mechanical factors to receptor–ligand binding and discuss the mechanisms underlying the cellular behavior mediated by these interactions, with particular emphasis on their biomedical implications. These findings not only enrich our understanding of various physiological and pathological processes from the molecular level, but provide potential clues for the development of practical therapies for relevant diseases. For instance, as VWF plays a prominent role in the shear-rate-dependent platelet adhesion in thrombus, agents targeting VWF interaction with the vessel wall or platelets could potentially help to prevent coronary artery disease [170,171,172].

In this review, we mainly focus on experimental investigations of mechanical-regulated receptor–ligand binding. In addition to experimental studies, theoretical and numerical modeling has become an attractive means and provided important enlightening information on receptor–ligand binding [173,174,175,176,177,178,179,180,181,182,183,184,185]. For example, Hu et al. performed theoretical and simulation studies to identify the receptor–ligand binding cooperativity resulting from thermal membrane fluctuation. This finding provides a basis and direction for further investigation [173]. Subsequently, this binding cooperativity is experimentally confirmed by Steinkühler et al. [184]. Additionally, to uncover the mechanism regarding the effect of lipid raft on the receptor–ligand binding, modeling studies based on Monte Carlo simulations of a mesoscopic model have been developed [175,176,177,178,179,180,181,182,183,185]. Their results, consistent with experimental observation, uncover and validate the cooperative effect of lipid raft and the entropic force induced by membrane fluctuation on the receptor–ligand binding, and provide important information and insight for understanding the role of raft microdomain in cell communication. Results from numerical modeling can also provide detailed information regarding atomic structures and dynamics and contribute to pharmaceutical development [174]. Integrating the experimental and computational methods will undoubtedly further lead to more fruitful achievements and enrich our understanding.

Last but not least, cells are exposed to microenvironments in vivo with multiple mechanical stimuli [186]. To fully understand how the receptor–ligand binding responds to these multiple mechanical stimuli, it is necessary to conduct studies that examine the coupling effects of two or more types of mechanical factors. Overall, our work will help researchers gain an overview of the area along with a deeper understanding of the receptor–ligand binding and provide some useful guidance for further research.

## Figures and Tables

**Table 1:** Relevant studies regarding the effect of tensile force on receptor–ligand binding.

Biomedical Implications	Type	Molecules	Tensile Force	Author [Reference]
Thrombosis	Slip bond	Integrin α_IIb_β_3_ and fibrinogen	~5–50 pN	Litvinov et al. [70]
Immune responses	Catch bond	NKG2D and different ligands	5, 10, and 15 pN	Fan et al. [72]
Thrombosis		Integrin α_5_β_1_ and fibronectin	—	Strohmeyer et al. [76]
Tissue formation and wound healing	Catch-slip bond	E-cadherin	0–50 pN; 0–70 pN	Rakshit et al. [69]
Immune responses		β_3_ integrin and Kindlin2	0, 20, 40, and 60 pN	Zhang et al. [79]
Inflammatory response and hemostasis		Mac-1and GPIbα	0, 25, 50, and 75 pN	Jiang et al. [80]
Cell motility	Cyclic mechanical reinforcement	Integrin α_5_β_1_ and fibronectin	Peak force < 50 pN	Kong et al. [81]
—		Integrin α_5_β_1_ and fibronectin	Peak force < 40 pN	Li et al. [82]

**Table 3:** Relevant studies regarding the effect of stretch on receptor–ligand binding.

Biomedical Implications	Molecules	Stretching Frequency and Magnitude	Author [Reference]
Cell reorientation	Adhesive receptors and ligands	10% stretch at 0.001, 0.05, 0.2, and 1 Hz; 1%, 2%, 4%, and 10% stretch at 1 Hz	Qian et al. [132]
	Integrin	0.01, 0.1, 1, 2, and 10 Hz	Kong et al. [133]
	Integrin	1–9% stretch; 0.1–10 Hz	Chen et al. [135]

**Table 4:** Relevant studies regarding the effect of compressive force on receptor–ligand binding.

Biomedical Implications	Type	Molecules	Compressive Force	Author [Reference]
—	Slip bond	—	0–20 × 10^−4^ pN/nm^2^	Xu et al. [140]
Inflammatory response	Catch bond	E-selectin and sialyl Lewis^a^	6–46 pN	Snook and Guilford [143]
Thrombotic response related to diabetes		Integrin α_IIb_β_3_ and fibrinogen	5–40 pN	Ju et al. [145]
Inflammatory response	Catch-slip bond	E-selectin and sialyl Lewis^a^	6, 12, 18, 30, 39 pN	Snook and Guilford [144]

## Data Availability

Not applicable.
